# Clinical Outcomes and Safety of Meropenem–Colistin versus Meropenem–Tigecycline in Patients with Carbapenem-Resistant *Acinetobacter baumannii* Pneumonia

**DOI:** 10.3390/antibiotics10080903

**Published:** 2021-07-23

**Authors:** Jae-Min Park, Kyung-Sook Yang, You-Seung Chung, Ki-Byung Lee, Jeong-Yeon Kim, Sun-Bean Kim, Jang-Wook Sohn, Young-Kyung Yoon

**Affiliations:** 1 Division of Infectious Diseases, Department of Internal Medicine, Korea University College of Medicine, 145 Anam-ro, Seongbuk-gu, Seoul 02841, Korea; jemin2653@gmail.com (J.-M.P.); paramentor@gmail.com (Y.-S.C.); anthromeda2017@gmail.com (K.-B.L.); agisae92@gmail.com (J.-Y.K.); puppybin@gmail.com (S.-B.K.); jwsohn@korea.ac.kr (J.-W.S.); 2 Department of Biostatistics, Korea University College of Medicine, 145 Anam-ro, Seongbuk-gu, Seoul 02841, Korea; myksyang@gmail.com

**Keywords:** *Acinetobacter baumannii*, meropenem, tigecycline, colistin

## Abstract

This study compared the clinical outcomes and safety of meropenem–colistin versus meropenem–tigecycline in the treatment of adult patients with carbapenem-resistant *Acinetobacter baumannii* (CRAB) pneumonia. A retrospective observational study of patients with CRAB pneumonia was performed at a 1048-bed university-affiliated hospital in the Republic of Korea between June 2013 and January 2020. All adult patients initially treated with meropenem–colistin were compared with those treated with meropenem–tigecycline to evaluate in-hospital mortality and adverse events. Altogether, 66 patients prescribed meropenem–colistin and 24 patients prescribed meropenem–tigecycline were included. All patients had nosocomial pneumonia, and 31.1% had ventilator-associated pneumonia. The minimum inhibitory concentrations of meropenem ≤ 8 μg/mL and tigecycline ≤ 2 μg/mL were 20.0% and 81.1%, respectively. The in-hospital and 28-day mortality rates were 40% and 32%, respectively. In the Cox proportional hazard regression analysis, predictors associated with in-hospital mortality included procalcitonin ≥ 1 ng/mL (adjusted hazard ratio (aHR), 3.39; 95% confidence interval (CI) 1.40–8.19; *p* = 0.007) and meropenem–colistin combination therapy (aHR, 2.58; 95% CI, 1.07–6.23; *p* = 0.036). Episodes of nephrotoxicity were significantly more common in the meropenem–colistin group than in the meropenem–tigecycline group (51.5% vs. 12.5%, *p* = 0.001). Meropenem–tigecycline combination therapy might be a valuable treatment option for patients with CRAB pneumonia.

## 1. Introduction

Carbapenem-resistant *Acinetobacter baumannii* (CRAB) has been listed as a critical priority pathogen by the World Health Organization’s 2017 global priority list of antibiotic-resistant bacteria; these bacteria require the development of new antibiotics [1]. *A. baumannii* is a prevalent etiologic agent causing diverse nosocomial infections and whose resistance to carbapenem is remarkably high—95% in some parts of the world and 85% in the Republic of Korea [2,3]. In particular, it is the major pathogen isolated from hospital-acquired pneumonia (HAP) and ventilator-associated pneumonia (VAP), with an imipenem resistance rate of 67.3% in Asian countries [4].

Currently, there are very few treatment strategies for CRAB pneumonia because CRAB has become resistant to most available antibiotics. Colistin is often the last treatment option for CRAB pneumonia, based on antimicrobial susceptibility tests. Unfortunately, colistin is associated with a high possibility of nephro- and neurotoxicity. Additionally, poor pulmonary penetration and the development of heteroresistance are major concerns. Due to the limited therapeutic options, tigecycline has also been prescribed for the treatment of CRAB pneumonia in clinical practice. It has been proven to be active in vitro against CRAB isolates, for which it has high susceptibility, and it is concentrated in the lung parenchyma in animal models [5,6]. Furthermore, there is no need to adjust the dose in patients with decreased renal function, and higher than standard doses can be utilized if necessary [7]. However, a previous study reported that patients treated with tigecycline for pneumonia showed significantly lower cure rates than those treated with imipenem [8]. A comparison analysis from the US Food and Drug Administration suggested that tigecycline therapy showed a higher associated mortality compared with other antibiotics in patients with VAP [9].

Given the limitations of monotherapy for CRAB pneumonia, antibiotic combination therapy has been explored as an option to improve clinical outcomes. The rationale for the use of combination therapy against CRAB pneumonia is based on the hypothesis that each antibiotic often interacts synergistically to increase pathogen killing, and it allows for the use of lower doses of antibiotics with a reduction in side effects; this can potentially help prevent the development of antibiotic resistance. However, optimal regimens for antibiotic combinations with maximum efficacy and safety remain challenging.

To the best of our knowledge, this study is the first to compare the clinical outcomes and safety of meropenem–colistin versus meropenem–tigecycline in the treatment of patients with CRAB pneumonia.

## 2. Results

### 2.1. Patients and Clinical Characteristics

A total of 535 consecutive non-duplicate patients with CRAB isolated from respiratory specimens were included in this study. Patients who initially received other adequate antibiotics, except meropenem–colistin or meropenem–tigecycline (n = 188), were also excluded. Patients who had polymicrobial pneumonia (n = 128), infections from other sources (n = 121), or meropenem–colistin or meropenem–tigecycline administered for less than three days (n = 8) were also excluded from the analysis. Finally, 90 patients treated with meropenem–colistin (n = 66) or meropenem–tigecycline (n = 24) as the initial effective antibiotics for at least three days were analyzed.

The demographic and clinical characteristics of the 90 patients are shown in Table 1. All of them had nosocomial infections, and 28 patients (31.1%) had VAP. Of these, 59 (65.6%) were men. The median age and median Charlson comorbidity index were 70 years (interquartile range (IQR]), 62–80 years) and 2 (IQR, 1–3), respectively. Sixty-eight patients (75.6%) had septic shock, and 12 patients (13.3%) had bacteremia caused by CRAB. In the univariate analysis, there were no significant differences in sex and comorbidities between the meropenem–colistin and meropenem–tigecycline treatment groups. The meropenem–tigecycline group was older than the meropenem–colistin group and had a significantly longer hospital stay before the onset of CRAB pneumonia than the meropenem–colistin group. Patients in the meropenem–colistin group were significantly more likely to have underlying malignancy and VAP than those in the meropenem–tigecycline group.

The minimum inhibitory concentration (MIC) of colistin for all isolates was ≤2 µg/mL. The distributions of tigecycline MIC ≤ 2 µg/mL and meropenem MIC 8 µg/mL were not significantly different between the two groups (Table 1).

### 2.2. Treatment Outcomes

The treatment outcomes of the 90 patients are presented in Table 2. The overall in-hospital mortality rate was 44%. The 14- and 28-day mortality rates were 28.9% and 35.6%, respectively. In the univariate analysis, there were no significant differences in clinical outcomes between the two treatment groups when assessing 14- and 28-day mortality, in-hospital mortality, and total length of hospital stay after a CRAB pneumonia diagnosis (Table 2).

Nephrotoxicity episodes were significantly more common in the meropenem–colistin group than in the meropenem–tigecycline group. Nephrotoxicity occurred within a median of seven days (IQR, 6–10 days) after colistin administration. There were no significant differences in the episodes of hepatotoxicity or nausea between the two groups (Table 2). Notably, there was no difference in the number of patients requiring a change in initial antibiotic therapy between the groups, and the duration before antibiotic change showed significant differences between the two groups (Table 2). The initial antibiotic regimen was changed due to disease progression (n = 5), renal toxicity (n = 5), and convulsions or their potential (n = 2) in the meropenem–colistin group and due to disease progression (n = 2) and hepatotoxicity (n = 1) in the meropenem–tigecycline group. Antibiotic regimen changes in the meropenem–colistin group (n = 12) included colistin monotherapy (n = 8; nebulizer therapy, n = 3), meropenem–tigecycline (n = 2), meropenem–ampicillin/sulbactam (n = 1), and colistin–ampicillin/sulbactam (n = 1). Those in the meropenem–tigecycline group (n = 3) comprised meropenem–ampicillin/sulbactam (n = 1), tigecycline–amikacin (n = 1), and tigecycline–colistin (n = 1).

Table 3 shows the comparisons of in-hospital mortality among patients with CRAB pneumonia who received different antibiotic regimens. In the Cox proportional hazards regression analysis, predictors associated with in-hospital mortality included procalcitonin ≥1 ng/mL (adjusted hazard ratio (aHR), 3.39; 95% confidence interval (CI) 1.40–8.19; *p* = 0.007) and meropenem–colistin combination therapy (aHR, 2.58; 95% CI, 1.07–6.23; *p* = 0.036) (Table 4).

**Figure 1:**
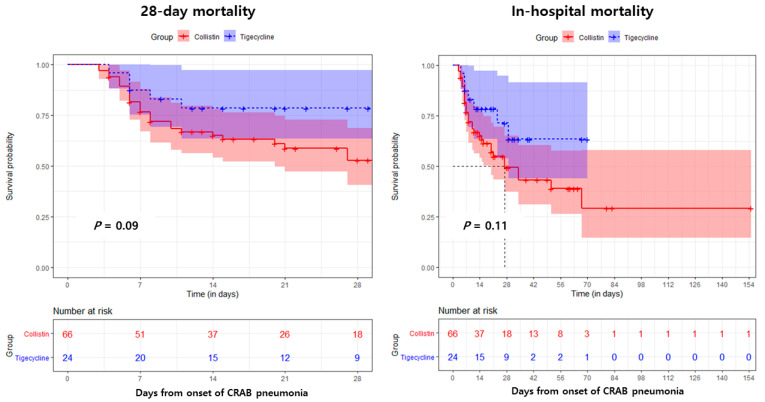
Kaplan–Meier survival curves for the univariate model comparing the meropenem–colistin and meropenem–tigecycline groups in patients with carbapenem-resistant *Acinetobacter baumannii* (CRAB) pneumonia.

**Figure 2:**
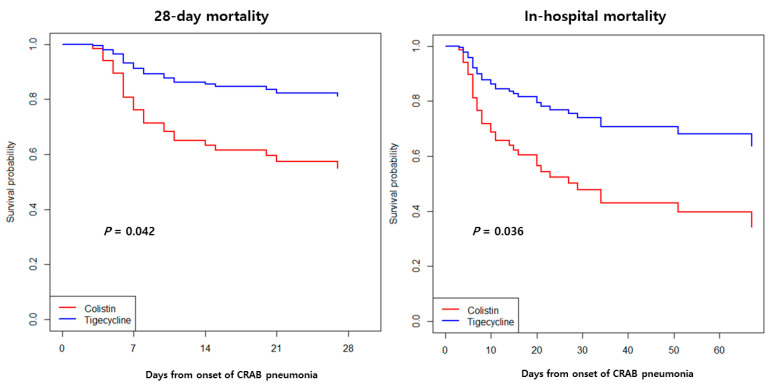
Kaplan–Meier survival curves for the multivariate model comparing the meropenem–colistin and meropenem–tigecycline groups in patients with carbapenem-resistant *Acinetobacter baumannii* (CRAB) pneumonia.

The Kaplan–Meier survival curve analysis showed that the associated in-hospital mortality of patients receiving meropenem–tigecycline therapy was lower than that of patients receiving meropenem–colistin therapy, although the difference was not significant (Figure 1). In contrast, using the Cox proportional hazards regression model for multivariate analysis, the cumulative survival curves were significantly different between the meropenem–tigecycline and meropenem–colistin groups (Figure 2).

In the subgroup analysis of patients in the meropenem–tigecycline group, no significant difference was found in 28-day mortality (4/19 (21.1%) vs. 1/5 (20.0%), *p* = 1.000) or in-hospital mortality (5/19 (26.3%) vs. 2/5 (40.0%), *p* = 0.608) between the tigecycline MIC ≤ 2 µg/mL and tigecycline MIC > 2 µg/mL groups. In the subgroup analysis for patients with VAP, there was no significant difference in 28-day mortality (11/25 (44.0%) vs. 0/3 (0%), *p* = 0.258) or in-hospital mortality (13/25 (52.0%) vs. 0/3 (0%), *p* = 0.226) between the meropenem–colistin and meropenem–tigecycline groups.

## 3. Discussion

This retrospective study showed that the combination of meropenem–tigecycline was more effective in reducing in-hospital mortality and nephrotoxicity in nosocomial CRAB pneumonia than meropenem–colistin. Multivariate analysis demonstrated that procalcitonin ≥ 1 ng/mL and meropenem–colistin combination therapy were significant predictors of in-hospital mortality among patients with CRAB pneumonia.

There is insufficient data regarding the steady-state pharmacokinetics and pharmacodynamics (PK/PD) of colistin in patients with CRAB infections. In complex cases, wherein the patient is in a fragile physiological state, pulmonary and plasma concentrations of colistin might be suboptimal; however, there is a high degree of inter-individual variability [10,11]. In addition, the high incidence of nephrotoxicity (reaching 60%) among those receiving colistin hinders dose escalation [12,13]. Notably, the heteroresistance observed frequently in multidrug-resistant *A. baumannii* supports the hypothesis that colistin therapy and extended interval dosage regimens may cause intractable problems in patients with CRAB infections [14]. These concerns have led physicians to consider better treatment options [11]. In our study, the degree to which nephrotoxicity contributed to mortality is unknown, although additional studies are needed to determine the optimal dose of colistin or ideal colistin-based regimens in patients with nosocomial pneumonia.

A recent meta-analysis found that tigecycline-based therapy was associated with higher in-hospital mortality in patients with CRAB pneumonia compared with that of colistin-based therapy [15]. Some studies have also disfavored tigecycline-based therapy for CRAB pneumonia [16,17]. However, these studies neither differentiated between monotherapy and therapy with tigecycline in combination nor analyzed the clinical efficacy of simultaneous administration of various antibiotics as part of combination therapy regardless of the microbiological properties. Meanwhile, a recent study demonstrated good clinical efficacy of salvage therapy comprising tigecycline and a prolonged infusion of imipenem/cilastatin in patients with VAP even with CRAB bacteremia [18]. Furthermore, it showed in vitro synergism or additivity of tigecycline plus imipenem/cilastatin against CRAB isolated from patients with VAP [18]. Several studies have suggested that tigecycline is a potential option for treating pneumonia caused by CRAB isolates, particularly with tigecycline MIC ≤ 2 μg/mL [16,19,20,21]. However, tigecycline has a bacteriostatic mechanism of action against CRAB isolates, and low serum levels are due to extensive and rapid distribution from the blood into tissues [22]. Breakthrough infection and the development of antibiotic resistance have raised concerns [23]. There have been attempts to overcome these shortcomings by increasing the daily dosage used for treating CRAB pneumonia, although clinical data are lacking [24,25]. In our study, favorable outcomes of the meropenem–tigecycline group may be related to the high proportion (81.1%) of CRAB isolates with tigecycline MIC ≤ 2 μg/mL and difference in VAP proportions between the two groups.

Since monotherapies are limited, many physicians prefer the use of combination therapy for CRAB pneumonia. For the treatment of diverse CRAB infections, carbapenems such as imipenem, meropenem, or doripenem are still presumed to be important components of a combination antibiotic regimen, even though this may appear counter-intuitive [26,27]. A previous study suggested that the efficacy of combination therapy containing carbapenems may be maximized when the meropenem MIC is ≤8 µg/mL, and it can be administered as a high-dose or prolonged infusion regimen to attain favorable PK/PD parameters [28,29]. Unfortunately, 80% of CRAB isolates in our study had meropenem MICs > 8 µg/mL, and meropenem was administered as a standard dose in a prolonged infusion regimen of 3 h.

This study included nosocomial pneumonia caused by CRAB and found an in-hospital mortality rate of 44.4%, somewhat higher that the mortality rates reported in previous studies (between 27% and 55%) [16,19,21]. In this study, 97.8% of participants were treated in the intensive care unit (ICU), 75.6% developed septic shock, and 31.1% had VAP. These characteristics may have contributed to the relatively high mortality rates. In our study, the association of a serum procalcitonin level ≥ 1 ng/mL with in-hospital mortality was comparable to findings observed elsewhere [21].

Our study has some limitations. First, it was a retrospective, monocentric design comprising a small population. Thus, there may have been some bias during data collection, and our findings may be limited in their application to other hospitals with different local epidemiology. Due to the small sample size, this study has insufficient statistical power to detect differences in clinical outcomes. Second, the measurement of the PK/PD parameters associated with colistin or tigecycline were not included in the study design. Although considerable variations in antibiotic concentrations existed for each patient with CRAB pneumonia, it is meaningful that the regimens used were those in real-life clinical settings. Third, a significant proportion of our patients received a miscellaneous regimen as salvage therapy, and there may have been unmeasured confounders. Fourth, the meropenem–colistin group included more patients with VAP than the meropenem–tigecycline group, although the Cox proportional hazard regression analysis was used to adjust for confounding factors. A previous study suggested that VAP was one of the predictors of 30-day mortality in patients with CRAB pneumonia treated with tigecycline [20].

## 4. Materials and Methods

### 4.1. Study Design and Patients

A retrospective single-center study was conducted in a 1048-bed university-affiliated hospital in the Republic of Korea between June 2013 and January 2020. The subjects included hospitalized adult patients (aged ≥ 18 years) with CRAB pneumonia who received either meropenem–colistin or meropenem–tigecycline for at least 3 days. For patients with multiple episodes of CRAB pneumonia, only the first episode was included in this analysis. Patients with other concurrent infections or polymicrobial infections of the respiratory tract were excluded to solely evaluate the impact of each antibiotic regimen for CRAB pneumonia. Patients who received meropenem–colistin or meropenem–tigecycline for <3 days and those diagnosed with a lung abscess or empyema in the initial evaluation were excluded from the analysis.

A loading dose of colistin (300 mg colistin base activity) or tigecycline (100 mg) was administered intravenously during the first 12 h of therapy. For colistin, daily maintenance doses were calculated according to the patient’s renal function as per the recommended guidelines [10]. The clinician decided on a dose of 75 mg of nebulized colistin every 12 h for three patients (4.8%) in the meropenem–colistin group. Daily maintenance doses of tigecycline were 50 mg administered intravenously every 12 h, regardless of the patient’s renal function. Meropenem was administered by prolonged infusion over 3 h, with a loading dose of 1 g every 8 h on the first day, followed by daily maintenance doses adjusted according to the patient’s renal function [30].

During the study period, there were no other standardized interventions for the treatment of CRAB pneumonia. The choice of antibiotic regimen and treatment duration was at the discretion of the attending physician. Combination therapy with meropenem–colistin or meropenem–tigecycline has been used as a definitive therapy in patients with CRAB pneumonia.

The study protocol was approved by the Institutional Review Board (IRB) of Korea University Anam Hospital (No. 2020AN0173). The IRB granted a waiver for the requirement of informed consent because this was a retrospective study.

### 4.2. Variables and Definitions

A diagnosis of pneumonia was made if patients showed a radiographic infiltrate, consolidation, cavitation, or pleural effusion that was new or progressive, along with symptoms and signs compatible with pneumonia: new-onset fever (38 °C) or hypothermia (<35.5 °C), leukocytosis (white blood cell (WBC) count > 12,000 cells/mm^3^), leukopenia (WBC count < 4000 cells/mm^3^), increase in oxygen demand, and increase in amount or property change to purulent sputum [31]. Pneumonia caused by CRAB was defined as clinical evidence of pneumonia with sputum, bronchoalveolar lavage, or tracheal aspirate cultures positive for CRAB from 7 days before to 3 days after the first dose of meropenem–colistin or meropenem–tigecycline. Sputum and tracheal aspirate specimens showing at least 25 neutrophils and <10 epithelial cells per low-power field in Gram stains were needed to confirm a diagnosis. The etiologic agent of pneumonia was determined as a quantitative culture ≥10^4^ cfu/mL from bronchoalveolar lavage, a semi-quantitative culture of at least moderate growth from sputum, or a quantitative culture ≥10^5^ cfu/mL from tracheal aspirate.

Carbapenem resistance was defined as an MIC of ≥8 μg/mL for meropenem and imipenem, according to the breakpoints of the Clinical and Laboratory Standards Institute [32]. HAP was defined as pneumonia that occurred ≥48 h after admission that was not present at the time of admission. VAP was defined as pneumonia that occurred >48 h after endotracheal intubation. Nephrotoxicity and septic shock were defined as described in previous studies [16,33].

The primary endpoint was defined as in-hospital mortality, for which we were searching for associated independent predictors. The secondary outcomes included nephrotoxicity, hepatotoxicity, and length of hospital stay.

The following data were collected from a review of medical records: demographic characteristics, underlying comorbidities, Charlson comorbidity index [34], presence of septic shock, use of mechanical ventilation, care in the ICUs, laboratory findings, in-hospital mortality, antibiotic therapy, and microbiological data.

### 4.3. Microbiological Tests

Bacterial identification and antimicrobial susceptibility testing were performed using matrix-assisted laser desorption/ionization time-of-flight mass spectrometry (Bruker Daltonics, Bremen, Germany) and a MicroScan WalkAway 96 plus System (Siemens Healthcare Diagnostics, Berkeley, CA, USA), respectively. The susceptibility results were interpreted based on the standard criteria defined by the Clinical and Laboratory Standards Institute [32].

### 4.4. Statistical Analyses

Categorical variables were described using numbers (proportions), and comparisons between groups were assessed using Pearson′s chi-square test or Fisher′s exact test. Continuous variables were described using medians and IQRs. For between-group comparisons of continuous variables, the two-sample Student′s *t*-test or the Mann–Whitney U test were used as appropriate.

To determine the independent predictors of in-hospital mortality, a multivariate Cox proportional hazards regression model was used to investigate the impact of multiple independent predictors. Variables were used to build the multivariate model if they independently predicted in-hospital mortality at the 10% significance level in the univariate model. The Kaplan–Meier survival estimate was used to evaluate the difference in survival curves during hospitalization between the two groups receiving different antibiotic therapies.

All tests were two-tailed, and a *p*-value < 0.05 was considered statistically significant. All analyses were performed using IBM SPSS Statistics, version 20.0 (IBM, Armonk, NY, USA) and SAS 9.2 (SAS Institute, Cary, NC, USA).

## 5. Conclusions

In conclusion, combination therapy with meropenem and tigecycline showed significantly lower in-hospital mortality and nephrotoxicity than combination therapy with meropenem and colistin. Therefore, this study demonstrates that meropenem–tigecycline therapy may be a valuable treatment option for CRAB pneumonia. Further larger-scale investigations should focus on identifying targeted patient populations that can maximize the effectiveness of specific antibiotic regimens.

## Figures and Tables

**Table 1:** Comparison of demographic and clinical characteristics between the meropenem–colistin and meropenem–tigecycline groups in patients with carbapenem-resistant *Acinetobacter baumannii* pneumonia.

Characteristics	Total (n = 90)	Colistin-Based (n = 66)	Tigecycline-Based (n = 24)	*p*-Value
**Demographic variable**				
Median age, years (IQR)	70 (62–80)	68 (61–76)	79 (67–85)	0.003
Male sex, n (%)	59 (65.6)	46 (69.7)	13 (54.2)	0.170
**Variables from current admission**				
Median length of hospital stay before CRAB pneumonia diagnosis (IQR), days	15 (10–33)	14 (9–27)	23 (15–46)	0.013
BMI ≥ 25 kg/m^2^	19 (23.8)	17 (28.3)	2 (10.0)	0.132
Ventilator–associated pneumonia	28 (31.1)	25 (37.9)	3 (12.5)	0.021
**Comorbidities, n (%)**				
Cardiovascular disease	49 (54.4)	35 (53.0)	14 (58.3)	0.655
Neurologic disease	35 (38.9)	25 (37.9)	10 (41.7)	0.744
Malignant disease	22 (24.4)	20 (30.3)	2 (8.3)	0.032
Trauma	8 (8.9)	5 (7.6)	3 (12.5)	0.435
Renal disease	11 (2.2)	9 (13.6)	2 (8.3)	0.721
Hepatic disease	6 (6.7)	6 (9.1)	0	0.187
Pulmonary disease	6 (6.7)	6 (9.1)	0	0.187
Metabolic disease	36 (40.0)	28 (42.4)	8 (33.3)	0.436
Median Charlson comorbidity score (IQR)	2 (1–3)	2 (1–4)	2 (1–2)	0.253
**Antimicrobial susceptibility, n (%)**				
Meropenem MIC = 8 µg/mL	18 (20.0)	12 (18.2)	6 (25.0)	0.554
Tigecycline MIC ≤ 2 µg/mL	73 (81.1)	54 (81.8)	19 (79.2)	0.767
**Clinical severity, n (%)**				
CRAB bacteremia	12 (13.3)	11 (16.7)	1 (4.2)	0.710
ICU admission	88 (97.8)	65 (98.5)	23 (95.8)	0.464
Septic shock	68 (75.6)	50 (75.8)	18 (75.0)	0.941
Mechanical ventilator	80 (88.9)	61 (92.4)	19 (79.2)	0.123
Hemodialysis	28 (31.1)	24 (36.4)	4 (16.7)	0.074
ECMO	7 (7.8)	6 (9.1)	1 (4.2)	0.670
**Laboratory findings at time of CRAB pneumonia diagnosis, n (%)**				
Hemoglobin ≤ 10 mg/dL	86 (95.6)	63 (95.5)	23 (95.8)	1.000
Platelet ≤ 100,000/mm^3^	61 (67.8)	46 (69.7)	15 (62.5)	0.518
Bilirubin ≥ 3 mg/dL	23 (25.6)	18 (27.3)	5 (20.8)	0.536
Albumin ≤ 3 mg/dL	87 (96.7)	63 (95.5)	24 (100.0)	0.562
C-reactive protein ≥ 100 mg/L	79 (87.8)	59 (89.4)	20 (83.3)	0.475
Procalcitonin ≥ 1 ng/mL	54 (66.7)	38 (65.5)	16 (69.6)	0.727

Abbreviations: BMI, body mass index; CRAB, carbapenem-resistant *Acinetobacter baumannii*; ECMO, extracorporeal membrane oxygenation; ICU, intensive care unit; IQR, interquartile range; MIC, minimum inhibitory concentration.

**Table 2:** Comparison of clinical outcomes between the meropenem–colistin and meropenem–tigecycline groups in patients with carbapenem-resistant *Acinetobacter baumannii* pneumonia.

Characteristics	Total (n = 90)	Colistin-Based (n = 66)	Tigecycline-Based (n = 24)	*p*-Value
**Adverse events during treatment, n (%)**				
Nephrotoxicity	37 (41.1)	34 (51.5)	3 (12.5)	0.001
Hepatotoxicity	35 (38.9)	28 (42.4)	7 (29.2)	0.254
Nausea	9 (10.0)	6 (9.1)	3 (12.5)	0.696
**Change of initial antibiotic therapy, n (%)**				
Antibiotic change	15 (16.7)	12 (18.2)	3 (12.5)	0.751
Median time before change of initial antibiotics (IQR), days	9 (6–12)	8 (6–11)	11 (7–13)	0.033
**Clinical outcomes, n (%)**				
In-hospital mortality	40 (44.4)	33 (50.0)	7 (29.2)	0.079
14-day hospital mortality	26 (28.9)	21 (31.8)	5 (20.8)	0.309
28-day hospital mortality	32 (35.6)	27 (40.9)	5 (20.8)	0.078
Median length of hospital stay after CRAB pneumonia diagnosis (IQR), days	16 (7–31)	16 (7–30)	21 (10–32)	0.398
Median length of hospital stay (IQR), days	39 (25–64)	34 (24–61)	47 (31–82)	0.080

Abbreviations: CRAB, carbapenem-resistant *Acinetobacter baumannii*; IQR, interquartile range.

**Table 3:** Comparison of demographic and clinical characteristics between survivors and non-survivors in patients with carbapenem-resistant *Acinetobacter baumannii* pneumonia.

Characteristics	Total (n = 90)	Survivors (n = 50)	Non-Survivors (n = 40)	*p*-Value
**Demographic variable**				
Median age, years (IQR)	70 (62–80)	70 (61–80)	71 (62–79)	0.855
Male sex, n (%)	59 (65.6)	31 (62.0)	28 (70.0)	0.427
**Variables from current admission**				
Median length of hospital stay before CRAB pneumonia diagnosis (IQR), days	15 (10–33)	15 (9–27)	17 (11–37)	0.134
BMI ≥ 25 kg/m^2^	19 (23.8)	9 (20.5)	10 (27.8)	0.444
Ventilator-associated pneumonia	28 (31.1)	15 (30.0)	13 (32.5)	0.799
**Comorbidities, n (%)**				
Cardiovascular disease	49 (54.4)	26 (52.0)	23 (57.5)	0.603
Neurologic disease	35 (38.9)	23 (46.0)	12 (30.0)	0.122
Malignant disease	22 (24.4)	6 (12.0)	16 (40.0)	0.002
Trauma	8 (8.9)	6 (12.0)	2 (5.0)	0.292
Renal disease	11 (12.2)	6 (12.0)	5 (12.5)	1.000
Hepatic disease	6 (6.7)	2 (4.0)	4 (10.0)	0.400
Pulmonary disease	6 (6.7)	4 (8.0)	2 (5.0)	0.689
Metabolic disease	36 (40.0)	17 (34.0)	19 (47.5)	0.194
Median Charlson comorbidity score (IQR)	2 (1–3)	1.5 (1–3)	2 (1–4)	0.060
**Antimicrobial susceptibility, n (%)**				
Meropenem MIC = 8 µg/mL	18 (20.0)	9 (18.0)	9 (22.5)	0.596
Tigecycline MIC ≤ 2 µg/mL	73 (81.1)	43 (86.0)	30 (75.0)	0.185
**Antimicrobial regimen for CRAB pneumonia, n (%)**				
Colistin-based regimen	66 (73.3)	33 (66.0)	33 (82.5)	0.079
Tigecycline-based regimen	24 (26.7)	17 (34.0)	7 (17.5)	0.079
**Clinical severity, n (%)**				
CRAB bacteremia	12 (13.3)	6 (12.0)	6 (15.0)	0.677
ICU admission	88 (97.8)	49 (98.0)	39 (97.5)	1.000
Septic shock	68 (75.6)	31 (62.0)	37 (92.5)	0.001
Mechanical ventilator	80 (88.9)	43 (86.0)	37 (92.5)	0.502
Hemodialysis	28 (31.1)	12 (24.0)	16 (40.0)	0.103
ECMO	7 (7.8)	1 (2.0)	6 (15.0)	0.042
**Laboratory findings at time of CRAB pneumonia diagnosis, n (%)**				
Hemoglobin ≤ 10 mg/dL	86 (95.6)	47 (94.0)	39 (97.5)	0.626
Platelet ≤ 100,000/mm^3^	61 (67.8)	29 (58.0)	32 (80.0)	0.026
Bilirubin ≥ 3 mg/dL	23 (25.6)	9 (18.0)	14 (35.0)	0.066
Albumin ≤ 3 mg/dL	87 (96.7)	47 (94.0)	40 (100.0)	0.251
C-reactive protein ≥ 100 mg/L	79 (87.8)	42 (84.0)	37 (92.5)	0.334
Procalcitonin ≥ 1 ng/mL	54 (66.7)	24 (53.3)	30 (83.3)	0.004
**Clinical outcomes, n (%)**				
Nephrotoxicity	37 (41.1)	14 (28.0)	23 (57.5)	0.005
Hepatotoxicity	35 (38.9)	10 (20.0)	25 (62.5)	<0.001
Median total length of hospital stay (IQR), days	39 (25–64)	48 (27–71)	32 (19–56)	0.041

Abbreviations: BMI, body mass index; CRAB, carbapenem-resistant *Acinetobacter baumannii*; ECMO, extracorporeal membrane oxygenation; ICU, intensive care unit; IQR, interquartile range; MIC, minimum inhibitory concentration.

**Table 4:** Cox proportional hazards regression analysis of predictors associated with in-hospital mortality in patients with carbapenem-resistant *Acinetobacter baumannii* pneumonia.

Independent Variables	Hazard Ratio	95% Confidence Interval	*p*-Value
**Meropenem–colistin combination therapy**	2.58	1.07–6.23	0.036
**Procalcitonin** **≥ 1 ng/mL**	3.39	1.40–8.19	0.007

## Data Availability

The datasets generated and analyzed during the current study are available from the corresponding author on reasonable request.
